# *Mycoplasma pneumoniae*–Associated Bronchiolitis Obliterans Following Acute Bronchiolitis

**DOI:** 10.1038/s41598-017-08861-7

**Published:** 2017-08-16

**Authors:** Chengsong Zhao, Jinrong Liu, Haiming Yang, Li Xiang, Shunying Zhao

**Affiliations:** 1 Department of Respiratory Medicine, Beijing Children’s Hospital, National Center for Children’s Health, Capital Medical University, Beijing, 100045 People’s Republic of China; 2 Department of Allergy, Beijing Children’s Hospital, National Center for Children’s Health, Capital Medical University, Beijing, 100045 People’s Republic of China

## Abstract

The characteristics of *Mycoplasma pneumonia* (*M. pneumoniae*)-associated bronchiolitis obliterans (BO) are not well known. We retrospectively reviewed 17 patients with *M. pneumoniae*–associated BO. All patients had *M. pneumoniae*–associated acute bronchiolitis prior to the development of BO. In the acute bronchiolitis stage, all patients had fever and cough; six patients also had wheezing and dyspnoea. BO was diagnosed approximately 1.5–8 months later based on clinical manifestations and chest high-resolution computed tomography (HRCT) findings. All patients presented with wheezing and/or dyspnoea at the time of diagnosis of BO. HRCT findings included mosaic attenuation, pronounced air trapping, central bronchiectasis and emphysema, according to disease severity. Lung function tests revealed mild to severe airway obstruction. Fourteen of 17 patients had a greater than 12% increase in forced expiratory volume in 1 second values after taking salbutamol. All patients had positive allergy test results and family or personal history of atopic disease. Four patients had a history of asthma before *M. pneumonia* bronchiolitis. Asthma was diagnosed before, at the time of or after the diagnosis of BO in 11 cases. *M. pneumoniae*–associated BO may therefore develop following *M. pneumonia* bronchiolitis and overlap with asthma.

## Introduction

Bronchiolitis obliterans (BO) is a disease of chronic airflow obstruction. It is pathologically characterised by large airway bronchiectasis and bronchiole obstruction by inflammatory granulation tissue, which ultimately progress to fibrosis and scarring, leading to partial or total occlusion of the airway lumen. The prognosis of BO is greatly influenced by the timing of diagnosis and intervention. Therefore, it is important to understand the characteristic features and risk factors of BO so that early diagnosis and intervention can be made. To our knowledge, there have been only a few reported cases of *Mycoplasma pneumoniae*–associated BO. In this study, we reviewed 17 patients with *M. pneumoniae*–associated BO to assess the clinical features and risk factors of *M. pneumoniae*–associated BO in children.

## Methods

### Subjects

Seventeen patients at Beijing Children’s Hospital who were diagnosed with BO following *M. pneumoniae* infection between February 2010 and June 2015 were included in this study. Patients with cystic fibrosis, bronchopulmonary dysplasia, primary ciliary dyskinesia, immunodeficiencies, connective tissue disease and congenital heart disease were excluded. All studies were performed following the relevant guidelines and regulations of Beijing Children’s Hospital. The methods were carried out in accordance with the approved guidelines. The study was approved by the Medical Ethics Committee of Beijing Children’s Hospital, National Center for Children’s Health, China. The parents of all study participants gave both verbal and written informed consent before study enrolment.

### Methods

This was an observational and descriptive study. The medical records of all subjects were retrospectively reviewed. Data collected included clinical presentations, physical examinations, radiographic features and personal and family history of atopic disease. Allergen and lung function testing was performed.

### Laboratory tests

Allergens were detected using immunoblotting kits (AllergyScreen®, MediwissAnalytic GmbH, Moers, Germany) on a Rapid Reader device (Mediwiss). *Mycoplasma* antibody was detected using a latex-agglutination test (Fujirebio Inc., Tokoyo, Japan).

### Pulmonary function tests

Ventilation/flow curve and reversibility analysis were conducted when the patient reached 5 years of age using the MasterScreen™ system (Germany) according to the American Thoracic Society guidelines. Data collected included forced expiratory volume in 1 second (FEV_1_), forced vital capacity (FVC), forced expiratory flow at 25–75% of FVC (FEF_25–75%_), vital capacity (VC), inspiratory capacity (IC) and end residual volume (ERV).

## Results

### Acute bronchiolitis stage

**Figure 1:**
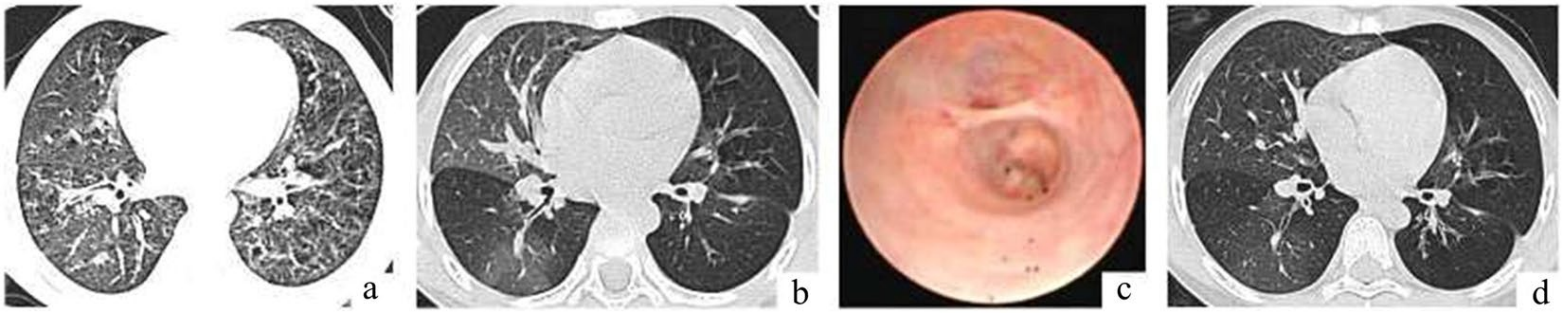
Representative HRCT and Bronchoscopy findings of a patient with moderate disease. (**a**) HRCT showing inflammatory bronchiolitis. (**b**) HRCT showing mosaic patterns, bronchiectasis and left lung hyperlucency. (**c**) Bronchoscopy showing a partially occluded lumen in segmental bronchi. (**d**) HRCT showing BO after 4 years of treatment.

**Figure 2:**
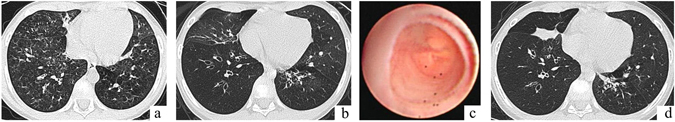
Representative HRCT and Bronchoscopy findings of a patient with severe disease. (**a**) HRCT showing inflammatory bronchiolitis. (**b**) HRCT showing mosaic patterns as well as marked bronchiectasis. (**c**) Bronchoscopy showing a completely occluded lumen in segmental bronchi. (**d**) HRCT showing BO after 4 years of treatment.

All patients had cough and fever; six patients also had wheezing and dyspnoea. Findings on chest high-resolution computed tomography (HRCT) were consistent with a diagnosis of acute inflammatory bronchiolitis, including centrilobular nodules, branching linear structures and ground-glass opacities (Figs 1a, 2a). Bronchial wall thickening was noted in 12 patients, with patchy or segmental consolidation in three cases. One case required mechanical ventilation and two cases under went continuous positive airway pressure (CPAP). The diagnosis of *M. pneumoniae* infection was based on the demonstration of an immunoglobulin M-specific anti-*Mycoplasma* antibody titre ≥1:320 or four-fold rising titre in acute and convalescent serum specimens. Blood, sputum and bronchoalveolar lavage fluid cultures as well as swab examinations were negative for bacteria and viruses in all children.

### BO development after acute bronchiolitis

BO was diagnosed based on clinical features, lung function testing and HRCT findings after an interval of 1.5–8 months (median 4.4 months) following acute *M. pneumoniae* bronchiolitis. At the diagnosis of BO, patients presented with wheezing and/or dyspnoea at rest or on exertion. Physical examination demonstrated fine rales, wheezing and retractions. BO was diagnosed early within 1.5–2 months following the onset of bronchiolitis in two patients. However, upon reviewing their HRCT findings, pronounced airtrapping and mosaic pattern was noted within 3 weeks following the onset of bronchiolitis in these two patients. In the remaining patients, BO was diagnosed 2–8 months later after *M. pneumoniae* bronchiolitis. Among 17 patients, five patients were initially diagnosed as having asthma and received inhaled corticosteroid treatment without improvement in lung function.

Severity of illness was graded as mild, moderate or severe as follows: mild illness included wheezing or tachypnoea with heavy physical activity and no desaturation on room air during sleep or physical activity (n = 5); moderate illness included wheezing or tachypnoea with moderate activities, normal oxygen saturation on room air during sleep and mild desaturation, i.e, <95% and >92% at sea level, with heavy activity (n = 6); severe illness included dyspnoea and retractions during sleep or with regular physical activityand desaturation to <92% at sea level with heavy activity(n = 6). The characteristics of all patients are shown in Table 1.

**Table 1:** Demographic and Clinical Features of 17 Patients with BO.

Age at diagnosis Gender	Clinical presentation	Imaging findings	Lung function(%)							Severity	Asthma, other personal atopic disease	Allergen	Family atopic disease	Follow up	
			FEV_1_			FVC		FEF_75%_							
			initial	latest	Increase% after salbutamol	initial	latest	initial	latest					Time (yrs)	Outcome
8yr 2mo M	Shortness of breath on exertion for 5mo after bronchiolitis	Mosaic attenuation	70.5	73.7	16.8	89.5	90.3	40.9	44.0	Mild	Asthma & AR before bronchiolitis	Dog fur, mould	Grand- father AR	1.0	Symptom and lung function improvement
3yr 4mo M	Wheezing on exertion for 2mo after bronchiolitis	Mosaic attenuation	71.7	74	16.5	88.9	92	30.4	34.8	Mild	Eczema&AR before bronchiolitis, Asthma after BO	Mould	Mother AR and urticaria	3.0	Symptom and lung function improvement
7yrs M	Wheezing on exertion for 2mo after bronchiolitis	Mosaic attenuation	72	80	25.6	98.2	100	44.6	50.5	Mild	Simultaneous Asthma &BO	Dust mite	Mother AR	1.5	Symptom and lung function improvement
4yrs 9mo M	Wheezing for 6mo after bronchiolitis	Mosaic attenuation	72.8	75.6	43	88.9	90	40.2	44	Mild	Eczema before bronchiolitis,Asthma after bronchiolitis	Dust mite, milk	Mother AR	2.0	Symptom and lung function improvement
7yrs 6mo M	Tachypnoea&wheezing for 6mo after bronchiolitis	Mosaic attenuation, bronchi-ectasis, air-trapping	63.6	71.7	13.2	85.2	96.2	18.4	24.4	Moderate	AR& atopic dermatitis, Asthma after bronchiolitis	Dust mite, pollen	Grand- father Asthma	1.5	Symptom and lung function improvement
4yrs F	Wheezing &dyspnoea for 2mo during & after bronchiolitis	Mosaic attenuation, bronchi-ectasis, air-trapping	50.9	63.0	22.1	81.0	87.3	15.6	24.4	Moderate	Eczema, Asthma after BO	Sea food	Father AR	5.5	Symptom and lung function improvement
3yrs 4mo M	Wheezing for 2mo during & after bronchiolitis	Mosaic attenuation, bronchi-ectasis, air-trapping	53	62.6	25	82.0	86.0	18.6	25	Moderate	Asthma &eczema before bronchiolitis	Mould, egg	Mother Atopic dermatitis	5.0	Symptom and lung function improvement
6yrs 4mo F	Wheezing &tachypnoea for 3mo after bronchiolitis	Mosaic attenuation, bronchi-ectasis, air-trapping	66.3	68.4	28.8	81.2	85.4	26.4	35.1	Moderate	AR &Asthma after BO	Shrimp	Mother & Father AR	4.0	Symptom and lung function improvement
5yrs 6mo M	Dyspnoeaon exertion for 5mo after bronchiolitis	Mosaic attenuation, air-trapping, left lung hyperlucent	57.3	58.9	2.8	84.6	85.5	18.3	19.1	Moderate	Asthma after bronchiolitis	Cock-roach, seafood	Mother Urticaria, Uncle Asthma	4.0	Symptom improvement, lung function no change
4yrs 4mo M	Tachypnoea on exercise for 6mo after bronchiolitis	Mosaic attenuation, left lung collapse	69.8	75	34	83	88	31.0	36	Moderate	AR before bronchiolitis, Asthma after BO	Dust mite	Father AR	2.0	Symptom and lung function improvement
7yrs 4mo M	Wheezing &dyspnoea for 6mo after bronchiolitis	Mosaic attenuation, bronchi- ectasis,air-trapping, left lung hyperlucent	37.4	35.1	32.5	55.2	55	11.9	10.9	Severe	Asthma before bronchiolitis	Dust mite	Mother Urticaria	1.0	Symptom improvement, lung function no change
3yrs 6mo F	Wheezing&dyspnoea for 1.5mo during and after bronchiolitis	Mosaic attenuation, central bronchi ectasis, emphysema	64.3	38	43	69.8	61.1	27.8	11.8	Severe	Simultaneou-s Asthma & BO, Urticaria& AR	Dust mite, pollen	Mother, aunt &grand- father AR &urticaria	5.0	Symptoms and lung function worse
4yrs 9mo F	Wheezing &dyspnoea for 8moduring &after bronchiolitis	Mosaic attenuation, bronchi- ectasis, air-trapping, left lung hyperlucent	48.4	50.8	16.2	77.3	78.2	14.6	13.9	Severe	Asthma after bronchiolitis	Dust mite, pollen, cat fur	Mother AR &asthma, Grand-father Asthma	6.0	Symptom improvement, lung function no change
5yrs M	Wheezing &dyspnoea for 7mo during & after bronchiolitis	Mosaic attenuation, central bronchi- ectasis, emphysema	48.1	33.8	81.8	77.3	62	15.7	13.8	Severe	Asthma before bronchiolitis	Sea food, dust mite	Mother AR	4.5	Symptoms and lung function worse
4yrs 6mo M	Wheezing &dyspnoea for 4mo during & after bronchiolitis	Mosaic attenuation, central bronchi-ectasis, emphysema	28.8	25.7	55.2	50.8	58	15.8	8.6	Severe	Eczema, Asthma after BO	Mould, Dust mite	Father AR	3.5	Symptoms and lung function worse
12yrs 8mo F	Dyspnoea for 4mo after bronchiolitis	Mosaic attenuation, central bronchi- ectasis, emphysema	17	18	9.8	49	49	10	8	Severe	Skin urticaria	Mould	Mother AR	1.0	Symptoms and lung function no change
9yr 2mo M	Wheezing on exertion for 3mo after bronchiolitis	Mosaic attenuation	74.3	75	1.9	78.5	80.7	40.5	38.9	Mild	Eczema	Dust mite	Father AR	1.0	Symptom improvement, lung function no change

Abbreviations: F, female; M, male; mo, month; BO, Bronchiolitis obliterans; AR, allergic rhinitis; FEV_1_, forced expiratory volume in 1 second; FVC, forced vital capacity; FEF, forced expiratory flow; yrs, years.

Chest HRCT at the time of BO diagnosis revealed a mild mosaic pattern bilaterally in five mild patients, notable airtrapping and minor bronchiectasis in four moderate patients (Fig. 1b), left lung collapse in one moderate patient and left lung hyperlucency in one moderate patient. Severe segmental bronchiectasis or central bronchiectasis and emphysema was noted in four severe patients (Fig. 2b), with pronounced air trapping, bronchiectasis and complete left lung hyperlucency in two severe patients. The involved areas on HRCT scans corresponded to those identified at the time of initial bronchiolitis. The HRCT findings did not change significantly in any patient after 1–6 years of follow-up (Figs 1d, 2d).

Four severe patients who underwent ventilation/perfusion scans had matched defects in ventilation and perfusion, even in the hyperlucent area.

Lung function testing at the time of BO diagnosis showed mild to severe airway obstruction and was repeated in every follow-up over 1–6years. The distribution of FVC%, FEV1%, and FEF_25–75%_ in initial and latest ventilation/flow curve analyses in the 17 patients are shown in Table 1. Of 17 patients, 14 demonstrated a complete reversible bronchodilator response, as indicated by a ≥12% increase in FEV_1_ following salbutamol inhalation(median, 27.5%; range, 1.9–81.8%) in every test. The latest complete reversible bronchodilator response is presented in Table 1. Reversible bronchodilator response became negative 1 year later, after diagnosis of BO in one patient.

Bronchoscopy demonstrated partial or complete obliteration of multiple segmental and sub-segmental bronchi in moderate and severe patients (Figs 1c and 2c), and poor ventilation in mild patients, predominantly in the basal sublingual segment of the left lower lobe.

### BO with atopic disease or asthma co-existing

All patients had positive allergen test results and personal and/or family histories of atopic disease. Four patients had asthma before *M. pneumoniae* infection. Asthma was diagnosed in 11 patients before, at the time of or after clinical diagnosis of BO based on episodic wheezing exacerbation related to common cold or exposure to allergens, rapid response to salbutamol inhalation and complete reversible post-bronchodilator response, together with personal and family histories of atopic disease and positive allergen test results.

### Outcomes

The patients were followed up for 1–6 years at 3–6-month intervals, or anytime when exacerbation occurred. Symptoms improved in all mild and moderate patients and two severe patients. Lung function improved in four of five mild patients and five of six moderate patients, remaining unchanged in one mild and one moderate patient, and the two severe patients whose symptoms improved. Symptoms and lung function worsened in three severe patients, and remained unchanged in one severe patient. The findings of HRCT did not change significantly in any patient. Mild patients could participate in normal activities with their peers, including physical activities. Exercise capacity was reduced to some extent in moderate patients. Severe patients required home oxygen therapy when pulse oxygen saturation (SpaO_2_) was <93%, leading to a poor quality of life. Thoracic deformity and clubbing developed in one severe patient, who had to drop out of school. During follow-up visits, readmissions occurred in two severe patients due to respiratory infections.

## Discussion

We herein describe 17 children who developed BO following *M. pneumoniae* bronchiolitis. Although lung biopsy has been considered the gold standard for the diagnosis of BO, lung biopsy does not always identify the characteristic lesions of BO due to its patchy distribution. The validity of HRCT in diagnosing BO has been well established because it is less invasive and demonstrates characteristic features^1–4^. We therefore did not perform lung biopsies. The clinical diagnosis of BO was made according to typical HRCT findings, clinical features and lung function test results. Interestingly, we found that BO coexisted with asthma in 15 of 17 cases. Since symptoms of dyspnoea and wheezing overlap between BO and asthma, mosaic pattern may be seen in asthmatic patients in acute exacerbation, while airtrapping, bronchial wall thickening and bronchiectasis may be observed in long-term uncontrolled asthmatic patients^5^, it is relatively difficulty that both asthma and BO could be simultaneously diagnosed in the same individual. However, asthma and BO are, in theory, distinct diseases that develop by unique mechanisms. In general, airflow was mainly limited by smooth muscle spasm in asthma, physiologically characterized by being reversible, either spontaneously or with treatment, with an episodic course whereas the airway of post-infection BO was mainly obstructed by inflammatory granulation tissue. The post-bronchodilator response in asthma shows complete reversibility of airway obstruction. In contrast, in BO, bronchodilator treatment provides either no reversibility or only partial reversibility of airway obstruction because of irreversible histological obstruction. In addition, in non-acute phase and controlled asthma, the HRCT findings were normal, but characteristic HRCT features were found in BO.

Asthma was diagnosed according to the evidence as described in the Results section in this study. We could exclude the effect of asthma on the findings of HRCT in all cases. Asthma was diagnosed in four cases before bronchiolitis. If airtrapping, bronchiectasis and mosaic pattern were influenced by asthma, these findings should be observed in the bronchiolitis stage, but in fact, were not observed in all patients, presented in Figs 1a and 2a. In a further 11 cases with asthma, episodic wheezing and exacerbation related to common cold or exposure to allergen stopped or decreased to once or twice a year without hospitalization after oral and/or inhaled steroid treatment, lung function returned to baseline levels or near to the level documented at time of diagnosis of BO. This suggests that asthma was well controlled, but the abnormal HRCT findings did not improve significantly. We therefore considered air-trapping, bronchiectasis and mosaic pattern to be representative of BO itself, not the influence of asthma. Moreover, bronchoscopy demonstrated partial or complete obliteration of multiple segmental and sub-segmental bronchi in moderate and severe patients, which are features of BO, further supporting a diagnosis of BO. Additional, evidence of BO was markedly decreased lung function, as presented in Table 1 and large and small airway obstruction remained after treatment.

Taken together, our patients shared features of both asthma and BO, indicating that these patients may have asthma–BO overlap syndrome. As only two of 17 cases did not have asthma, we could not compare BO in the 2 patients with BO in the 15 cases with asthma, but we have discussed these cases together.

BO is frequently preceded by lower respiratory tract infection in infants, and adenoviruses are the most commonly associated agents. There have been a few reports that M. pneumoniae infection can also cause BO^6–9^ including a report of unilateral hyperlucent lung syndrome in an 11-year-old girl^10^. Although pneumonitis has been the most common pathological characteristic in patients with M. pneumoniae infection, M. pneumoniae can also cause bronchiolitis^4, 11^. Chan *et al*. reported an adult case of M. pneumoniae–associated bronchiolitis caused severe restrictive lung disease^4^. Other case reports of M. pneumoniae–associated BO did not describe the type of M. pneumoniae infection.

We found that M. pneumoniae–associated BO developed following acute M. pneumoniae bronchiolitis in all cases in this study. It is well known that the main mechanism of BO development is related to airway epithelial injury and subsequent fibroblastic proliferation. Because M. pneumoniae infection may elicit airway epithelial injury and sloughing, it is plausible to consider that M. pneumoniae–associated BO may occur following acute M. pneumoniae bronchiolitis. This hypothesis is strongly supported by the report of Rollins *et al*.^12^, who observed five adult patients with open lung biopsy specimen-proven inflammatory (cellular) bronchiolitis due to M. pneumoniae. These five patients had extensive injury to the respiratory mucosa, loss of cilia and ciliated cells and fibrosis.

Kraft M and Martin R *et al*. reported that M. pneumoniae was present in the lower airways of chronic, stable asthmatics adults. It suggested that M. pneumoniae may play a role in the pathogenesis of chronic asthma^13^. We did not dynamically track persistent M. pneumoniae infections in this study, but did test bronchoalveolar lavage fluid in 3 of 17 patients by PCR at the time of BO diagnosis and no positive results were found.

Why does asthma–BO overlap happen in these patients. There are several reasons that asthma–BO overlap might occur, for example, the two conditions may share common risk factors or origins such as atopy; M. pneumoniae infection can trigger both asthma and BO; and importantly, both diseases can have common etiologic mechanisms, with airway epithelial injury playing an important role in asthma^14–16^ as well as BO.

## Conclusion

*M. pneumoniae*–associated BO may develop following *M. pneumonia* bronchiolitis. In children with M. pneumoniae bronchiolitis, if wheezing and/or dyspnoea do not resolve within the expected time frame and/or lung function does not improve following M. pneumoniae bronchiolitis, development of BO must be considered. *M. pneumoniae*–associated BO may subsequently overlap with asthma.
